# *Toxocara canis*-induced changes in host intestinal microbial communities

**DOI:** 10.1186/s13071-023-06072-w

**Published:** 2023-12-19

**Authors:** Soben Sieng, Ping Chen, Na Wang, Jing-Yun Xu, Qian Han

**Affiliations:** 1 Laboratory of Tropical Veterinary Medicine and Vector Biology, School of Life and Health Sciences, Hainan University, Haikou, 570228 Hainan People’s Republic of China; 2 One Health Institute, Hainan University, Haikou, 570228 Hainan People’s Republic of China

**Keywords:** *Toxocara canis*, Intestinal flora, *16S rRNA* high-throughput sequencing

## Abstract

**Background:**

*Toxocara canis* is a roundworm that resides in the gastrointestinal tract of dogs and causes various pathological changes. The dog’s intestinal system consists of a diverse and dynamic bacterial community that has extensive effects on intestinal physiology, immunity and metabolics. In the case of intestinal parasites, interactions with the host intestinal flora are inevitable during the process of parasitism.

**Methods:**

We studied the role of *T. canis* in regulating the composition and diversity of the intestinal flora of the host by high-throughput sequencing of the 16S ribosomal RNA gene and various bioinformatics analyses.

**Results:**

The α-diversity analysis showed that *Toxocara canis* infection resulted in a significant decrease in the abundance and diversity of host intestinal flora. The β-diversity analysis showed that the intestinal flora of infected dogs was similar to that carried by *T. canis*. Analysis of the microflora composition and differences at the phylum level showed that the ratio of Firmicutes to Bacteroidetes (F/B ratio) increased with *T. canis* infection. Analysis of species composition and differences at the genus level revealed that the proportion of some of the pathogenic bacteria, such as *Clostridium* sensu stricto and *Staphylococcus*, increased after *T. canis* infection.

**Conclusions:**

*Toxocara canis* infection affected the composition and diversity of the flora in the host intestinal tract. These results not only shed light on the potential mechanism of *T. canis* invasion and long-term survival in the intestinal tract, but also provide a new basis for the development of anthelmintic drugs.

**Graphical Abstract:**

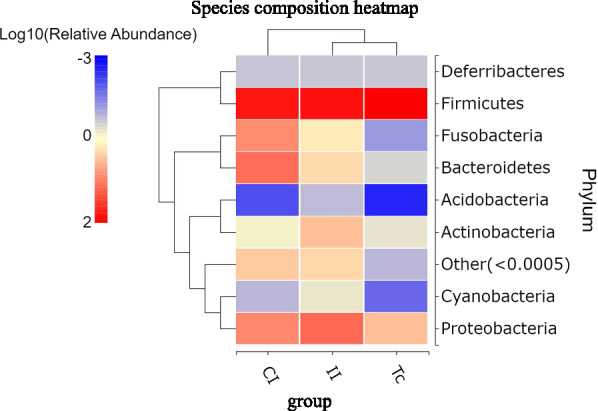

**Supplementary Information:**

The online version contains supplementary material available at 10.1186/s13071-023-06072-w.

## Background

*Toxocara canis* is a zoonotic helminth that is primarily prevalent in tropical regions and thought to infect over 100 million dogs and tens of millions of humans annually. By the year 2050, toxocariasis is predicted to rank among the most common parasitic diseases worldwide if cutting-edge scientific methods are not developed to combat *T. canis* infection [1]. *Toxocara canis* is one of the most significant gastrointestinal helminths in dogs and other canids, and the adults are usually parasitic in the small intestine of puppies [2, 3]. The intestinal system in vertebrates consists of a community of complex and constantly changing bacteria that play a crucial role in intestinal functions, immune responses and metabolic processes. Parasites have the ability to generate substantial changes to the physical structure of the intestine and influence the immune system [4]. They also interact with the diverse bacterial population present in the intestine [5], and these interactions can have a significant impact on the outcome of infections and influence the overall health and disease status of the host [6, 7]. On the one hand, the microbiota in the intestine has the ability to change the colonization, replication and virulence of parasites; on the other hand, the presence of parasites in the gastrointestinal tract can bring about significant changes to both the intestinal ecosystem and the environment where the microbiota resides [6, 8, 9]. Several studies have indicated that parasitic infections can influence the diversity of gut microorganisms [10–12].

To date, academic research has mainly focused on the impacts of *Trichuris trichiura* on the composition of the gut microbiota, followed by hookworm species and *Ascaris* spp. [13]. Easton et al. [14] not only elucidated the effects of *Trichuris* infection on the host gut microbiota but also further analyzed the changes in the microbiome of individuals co-infected with *Trichuris* and *Ascaris*. Through meta-analysis, Kupritz et al. [13] found that *Enterobius* and *Trichuris,* both of which parasitized in the large intestine, had a greater impact on intestinal microbial composition than other helminths, which parasitized in the small intestine (e.g. *Ascaris*, hookworm, *Strongyloides* and *Haplorchis taichui* [intestinal fluke]). However, analysis of the mechanisms by which *T. canis* regulates the host intestinal flora is still lacking. The aim of the present study is to examine how *T. canis* impacts the composition and diversity of the intestinal flora in dogs and, more importantly, to explore the correlation between the changes in host intestinal flora and the flora carried by *T. canis*. An understanding of the mechanisms and outcomes of interactions between parasites and flora can lay the basis for predicting the wider effects of specific anthelmintic and antibiotic treatments, as well as contributing towards the development of more efficient treatments for parasitic infections.

## Methods

### Animals and ethics statement

A total of six female dogs (5–6 months old; weight: 6–7 kg, body mass index: 21–23 kg/m^2^) were purchased from a farm in Haikou, Hainan Province for use in this experiment. Three of the dogs were found to have *T. canis* eggs in their feces; no *T. canis* eggs were found in their feces of the other three dogs. The dogs were housed in cages with free access to water and 150 g of food per day, and acclimatized to the experimental environment. The use of the dogs in this experiment was subjected to a thorough assessment and received official approval from the Hainan University Institutional Animal Care and Use Committee (HNUAUCC-2023-00201).

### Experiment grouping and sampling

All six dogs were euthanized and their small intestines dissected to collect the contents. *Toxocara canis* adults were found in the intestines of the three dogs which had been determined to have *T. canis* eggs in their feces; these dogs were designated the infected group (II group). No *T. canis* adults were found in the intestines of the other three dogs; these dogs were considered to be the control group (CI group). The collected *T. canis* adults (Tc group) were washed 3–5 times with normal saline. During the sampling procedure, care was taken that samples were not contaminated. After collection, each sample was placed into an appropriate sterile tube, which was then snap-frozen in a liquid nitrogen box.

### DNA extraction and purification

DNA was extracted from the samples using the QIAamp DNA Stool Mini Kit (Qiagen, Hilden Germany), and DNA concentration was measured using the Qubit Fluorometer Kit (Life Technologies, Thermo Fisher Scientific, Waltham, MA, USA). Sample quality was assessed by 1% agarose gel electrophoresis, following which the samples were analyzed by high-throughput sequencing to study the composition of the intestinal flora by amplifying the target region, specifically the V3–V4 regions of the 16S ribosomal gene.

###  High-throughput sequencing of 16S rRNA gene

PCR amplification was performed with 30 ng of genomic DNA (gDNA) and the corresponding fusion primers. The PCR amplification products were subsequently purified using Agencourt AMPure XP magnetic beads (Beckman Coulter, Inc., Brea, CA, USA), following which the purified products were dissolved in elution buffer, the labels attached and library construction completed. The fragment range and concentration of the library were measured on an Agilent 2100 Bioanalyzer (Agilent Technologies, Santa Clara, CA, USA). The qualified libraries were sequenced on the Ilumina Hiseq 2500 sequencing platform (Ilumina Inc., San Diego, CA, USA) according to the size of the inserted fragment. To ensure high-quality data, the raw data from sequencing underwent clipping and filtering processes to remove any low-quality reads. These valid reads were then merged into tags based on the overlap. A subsequent filtration step was used to extract the target segments.

### Bioinformatics analysis

To analyze the taxonomic composition of each sample, the sequences were grouped into operational taxonomic units (OTUs) based on a 97% sequence identity threshold. The Mothur algorithm was then used to assign a taxonomic classification to each OTU sequence, utilizing the SILVA 16S rRNA database (Version 128) https://www.arb-silva.de/documentation/release-128. Alpha and beta diversities were assessed using the Quantitative Insights Into Microbial Ecology (QIIME) database (http://qiime.org/1.4.0/). Linear discriminant analysis effect size (LEfSe) was employed to identify taxa that exhibited differences among groups, and linear discriminant analysis (LDA) was utilized to assess the impact of specific taxa. The Jensen-Shannon distance and the partitioning around medoids (PAM) algorithm were used to compute distances based on the relative abundance of taxa on the phylum or genus level. The optimal number of clusters (denoted as the *K*-value) was determined using the Calinski-Harabasz index. The results of the clustering analyses were visualized using between-class analysis or principal coordinates analysis.

### Statistical analysis

The statistical analysis was conducted using SPSS software version 20.0 (SPSS IBM Corp., Armonk, NY, USA). Hypothesis testing procedures followed a two-sided approach, providing measurements and corresponding *P* values. The threshold for determining significant differences was set at a *P*-value < 0.05. A one-way analysis of variance was used for further comparisons between groups.

## Results

###  High-throughput sequencing of 16S rRNA gene

**Fig. 1:**
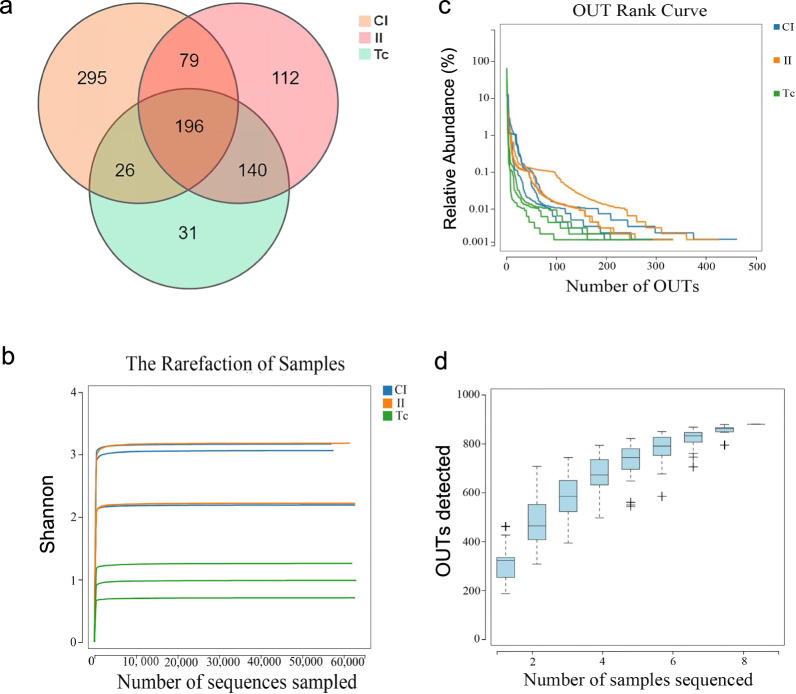
OTU cluster analysis. OTUs were generated according to 97% sequence similarity clustering. **a** Venn diagram of the unique and common OTUs among the three groups. **b** Rank-abundance curves. **c** Rarefaction curve. **d** Species accumulation curve. CI, Intestine samples of dogs in control group; II, intestine samples of dogs in infected group; OTU, operational taxonomic unit; Tc, *Toxocara canis* samples

16S rRNA genes isolated from the samples of intestinal contents dissected from the six dogs and three samples of *T. canis* were amplified by PCR and then subjected to high-throughput sequencing. Using a similarity threshold of 97%, the reads were grouped into OTUs, resulting in a total of 879 OTUs. An informative Venn diagram revealed that 596 of these 879 OTUs were in the CI group, 527 were in the II group and 393 were in the Tc group. Furthermore, 295 OTUs were unique to the CI group, 112 were unique to the II group and 31 were unique to the Tc group. Also, the II group shared 140 OTUs exclusively with the Tc group, excluding the OTUs shared between all three groups (Fig. 1a).

The rank-abundance curve in Fig. 1b demonstrated that the flora in the II group had lower species richness and evenness than the flora in the CI group, and that the Tc group had the lowest species richness and evenness of all three groups. The Shannon–Wiener index is a measure that evaluates the diversity of microorganisms in a specific sample, with higher values indicating greater community diversity. In this particular study, the curves for each group demonstrated a tendency towards flattening, suggesting that the amount of sequencing data obtained was adequate in terms of capturing most of the microbial information present in the samples from the three groups (Fig. 1c). The species accumulation curve was employed to estimate and predict the increase in species richness as the sample size grows within a community. The accumulation curve depicted in Fig. 1d, with the upward trend at the curve's end, indicates that the sample size was sufficient in terms of reflecting the species composition of the community.

### Diversity analysis

#### Alpha diversity analysis

**Fig. 2:**
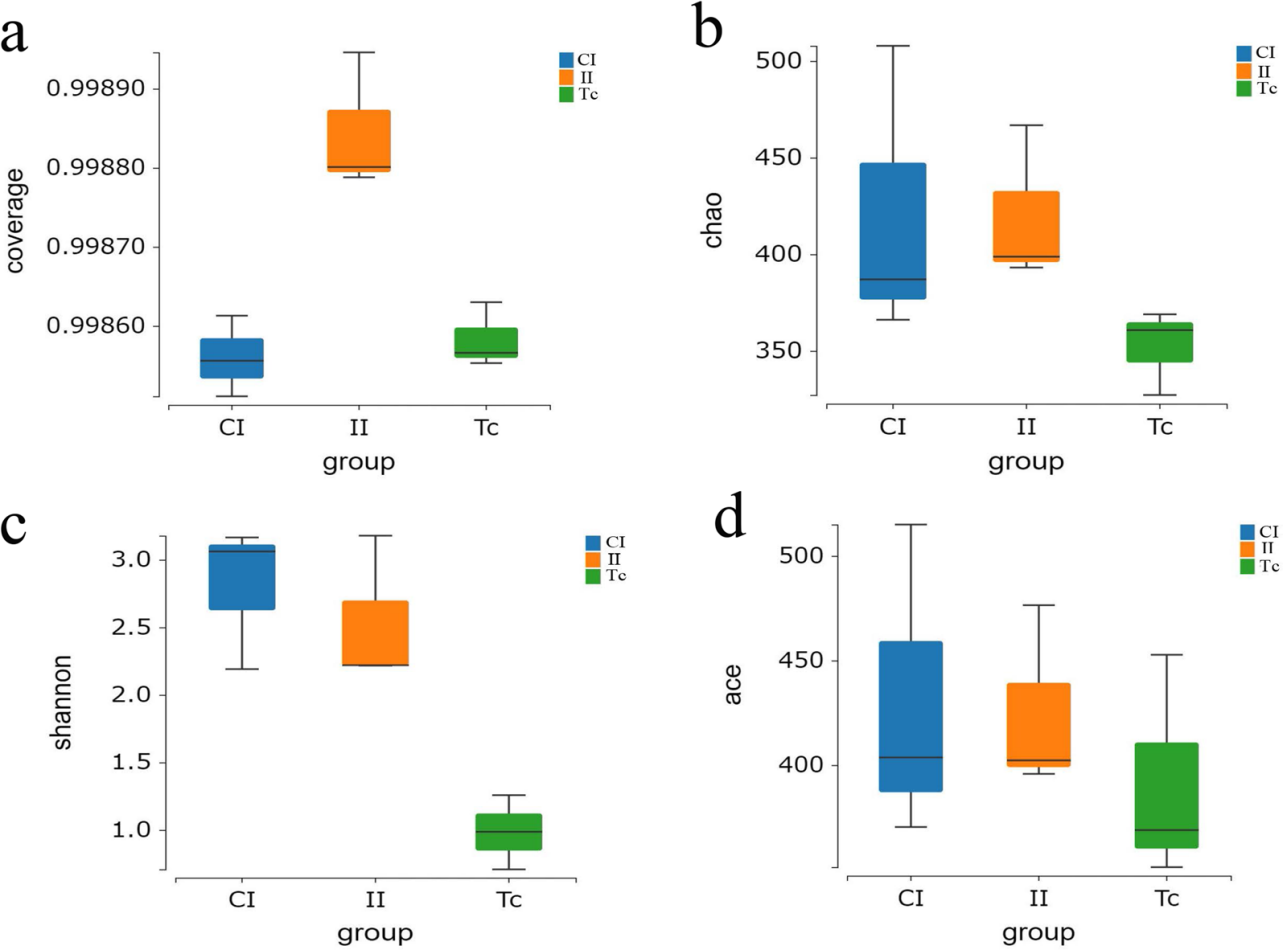
Boxplots showing comparisons of the alpha diversity indicators of flora among the three experimental groups. **a** Coverage index, **b** Chao index, **c** Shannon index, **d** Ace index. CI, Intestine samples of dogs in control group; II, intestine samples of dogs in infected group; Tc, *Toxocara canis* samples

To examine the variety of bacterial species in the CI, II and Tc groups, we assessed alpha diversity, and the results are shown in Fig. 2. Alpha diversity measures the number of distinct microbial taxa found in each sample, indicating both the abundance and distribution of the community. Our results showed that Good’s coverage index was > 95% for each group, indicating that the flora in all samples were effectively representative. The Ace, Chao and Shannon indices showed that the flora richness, diversity and evenness of the Tc group was lower than those of the CI and the II group, demonstrating that compared with the animal intestinal flora, the flora of the parasite had less richness and diversity.

#### Beta diversity analysis

**Fig. 3:**
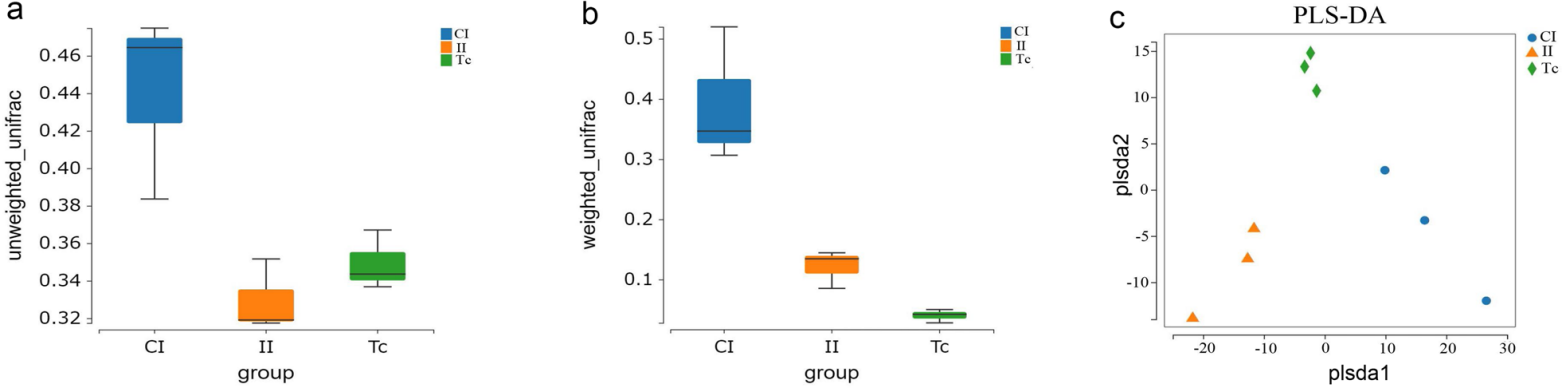
Comparison of beta diversity indicator of flora among CI, II and Tc groups. **a** Unweighted uniFrac analysis, **b** Weighted uniFrac analysis, **c** Partial least-squares discriminant analysis. CI, Intestine samples of dogs in control group; II, intestine samples of dogs in infected group; plsda, partial least-squares discriminant analysis; Tc, *Toxocara canis* samples

Beta diversity is the comparison of species presence, abundance and phylogenetic relationships among community members, and is expressed by calculating the similarity of microbial species between different samples. The evaluation metrics used in our investigation are the unweighted UniFrac distance metric and the weighted UniFrac distance metric. Both of these metrics indicated that the similarity in flora composition between the II and Tc groups was significantly higher than the similarity between the II and CI groups (Fig. 3a, b). The result of partial least squares-discriminant analysis (PLS-DA) showed that the CI, II and Tc groups each formed a different cluster, indicating that the sample grouping effect was good and the difference between groups was obvious (Fig. 3c).

#### LEfSe analysis

The LEfSe analysis was performed to discover significant biomarkers among the different groups. The results of the LEfSe analysis for the CI, II and Tc group, showing 75 biomarkers with a LDA score > 2, are displayed in Additional file 1: Figure S1 . There were seven biomarkers with a LDA score > 4. In the Tc group, *Clostridium *sensu stricto (*Clostridium *s.s.) and *Clostridiaceae* could serve as potential biomarkers, while in the II group, *Pseudomonas* and *Pseudomonadaceae* could serve as biomarkers. The LEfSe clustering analysis showed significant variations across multiple hierarchical levels between the three groups, as illustrated in Additional file 2: Figure S2.

### Analysis of species composition and difference

#### Species composition and difference analysis at the phylum level

**Fig. 4:**
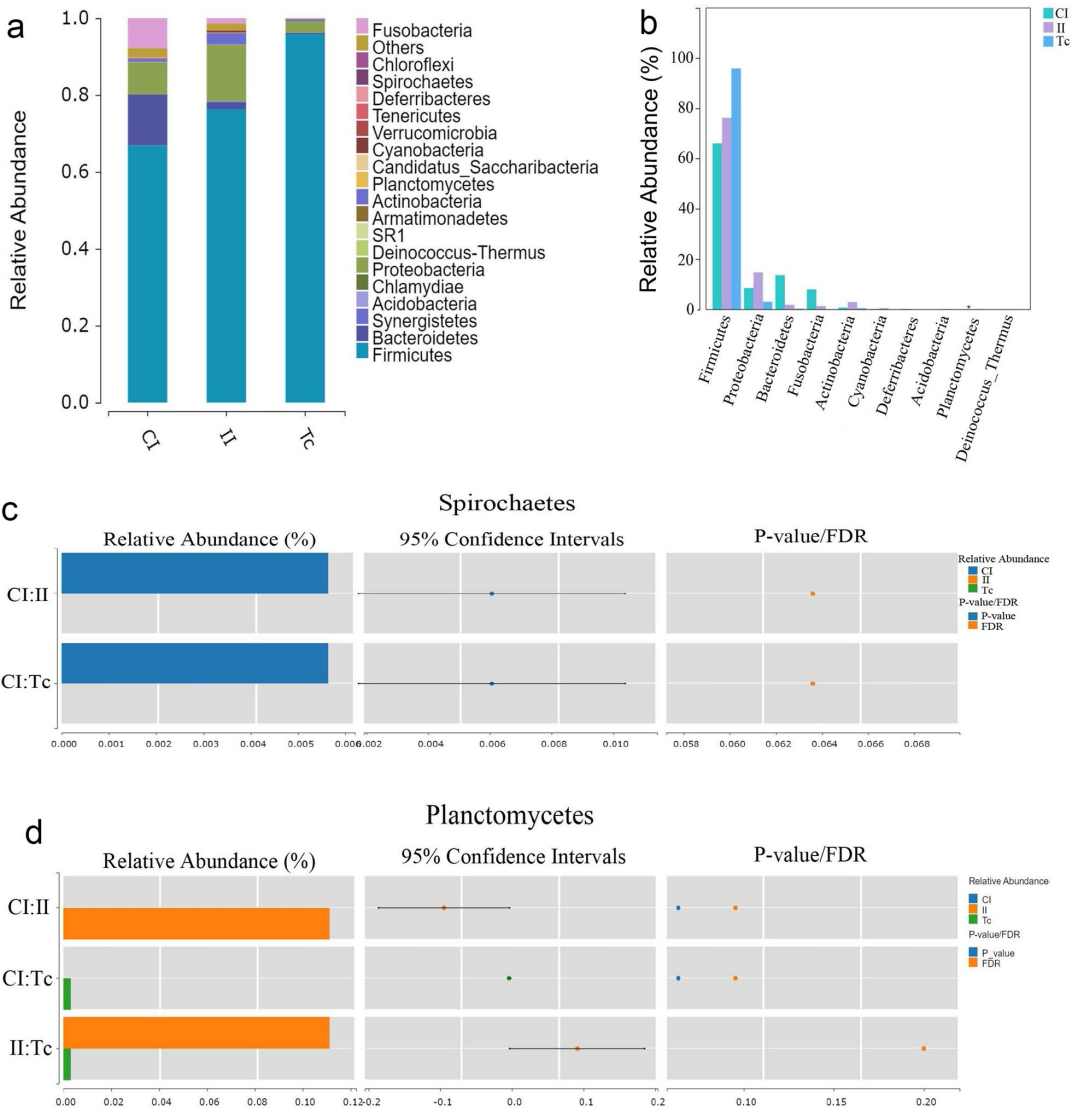
Differences in species composition of flora in the CI, II and Tc groups at the phylum level.** a** The horizontal coordinate is the experimental group, and the vertical coordinate is the relative abundance of the species annotated to. RDP Classifer (version 1.9.1) software was used to compare the OTU representative sequence with the database for species annotation, and the confidence threshold was set to 0.6. Species not annotated at this taxonomic level and whose abundance was < 0.5% of the sample were combined as “Others”.** b** The top 10 species according to phylum level in the CI, II and TI groups. Note that the significance of the test of difference is marked with an asterisk at the top of the bar graph if available.** c**,** d** Differences in the Wilcox test results for Planctomycete (**c**) and for Spirochaetes (**d**). Left panel, histogram showing the relative abundance of each species within their respective groups; center panel, the log2 value of the average relative abundance ratio for the same species between the two groups; right panel, the *P*-value and FDR values obtained through a Wilcoxon test. If both the *P*-value and FDR values are < 0.05, there is a significant difference in abundance between the two groups for that species. CI, Intestine samples of dogs in control group; FDR, false discovery rate; II, intestine samples of dogs in infected group; Tc, *Toxocara canis* samples

As shown in Fig. 4a, the species composition histogram explained the dominant species and the relative abundance of each group. The dominant species (value of relative abundance > 0.05) in the CI group were mainly members of phyla Firmicutes, Bacteriodetes, Proteobacteria and Fusobacteria; the dominant species in the II group were mainly members of phyla Firmicutes and Proteobacteria; and the dominant species in the Tc group were mainly members of phylum Firmicutes. Although species of phylum Firmicutes were the dominant species in all three groups, this dominance was most significant in the Tc group. The difference in the abundance of flora at the phylum level among all groups is shown in Fig. 4b. Compared with the CI group, the community abundance of Firmicutes in the II and Tc group increased, indicating that the increase in the proportion of Firmicutes in the intestinal flora caused by *T. canis* infection might be due to the flora carried by *T. canis*. On the other hand, we found that the non-dominant Planctomycetes only appeared in the II and the Tc group, leading us to hypothesize that the infection of *T. canis* would cause the emergence of Planctomycetes in the intestinal flora of dogs and that Planctomycetes might come from *T. canis*. Also, Spirochaetes were significantly decreased after *T. canis* infection (Fig. 4c, d).

#### Species composition and difference analysis at the genus level

**Fig. 5:**
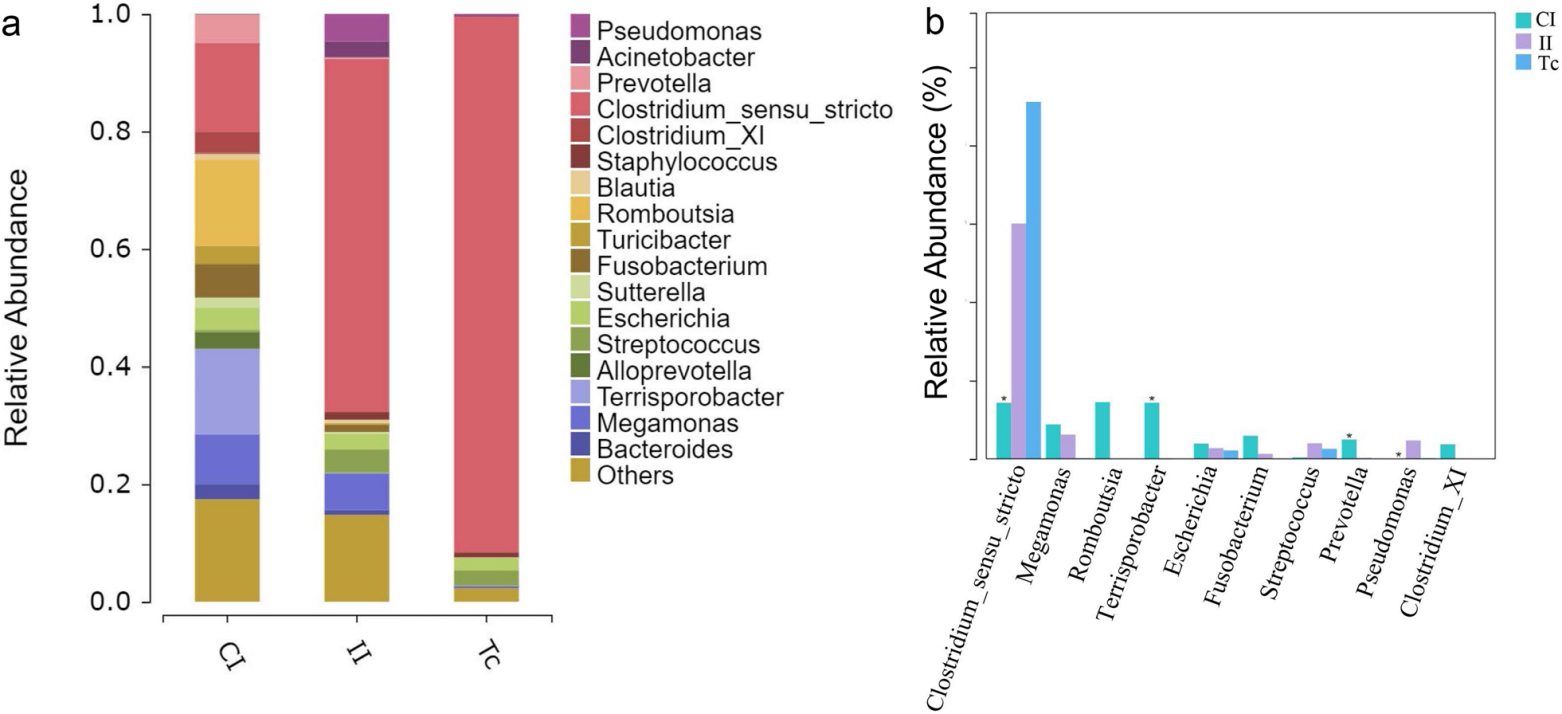
Differences in species composition of flora in the CI, II and Tc group at the genus level. The horizontal coordinate is the sample name and the vertical coordinate is the relative abundance of the species annotated. Species not annotated at this taxonomic level and whose abundance was < 0.5% of the sample were combined as “Others” (**a**). **b** The top 10 species at the genus level in the CI, II and TI group. Note that the significance of the test of difference was marked with an asterisk at the top of the bar graph if available. CI, Intestine samples of dogs in control group; II, intestine samples of dogs in infected group; Tc, *Toxocara canis* samples

A comparison of the changes in flora composition in the CI, II and Tc groups at the genus level (Fig. 5a) revealed that the dominant genera (value of relative abundance > 0.05) in the II group were *Clostruidium* s.s. and *Megamonas* and that *Clostruidium* s.s. was the only dominant genus in the Tc group. The differences in the abundance of flora at the genus level among all groups are shown in Fig. 5b. The relative abundance of *Clostruidium* s.s. was significantly increased in the II and the Tc groups compared with the CI group. The relative abundances of *Romboutsia*, *Terrisporobacter* and *Prevotella* were significantly decreased after infection with *T. canis*; in comparison, these three genera were almost undetectable in *T. canis*. On the other hand, the relative abundance of *Staphylococcus* was higher in the II and the Tc groups; in comparison, this genus was almost undetectable in the CI group (Fig. 5b). These results led us to hypothesize that infection by *T. canis* caused the increase of *Clostruidium* s.s. and the emergence of *Staphylococcus* in the intestinal flora of dogs, possibly from the flora carried by *T. canis*. In addition, *T. canis* infection significantly increased the proportion of harmful bacteria, such as *Akkermansia*, *Anaerococcus*, *Corynebacterium*, *Craurococcus*, *Frederiksenia*, *Haemophilus* (Additional file 3: Fig. S3)*,* and significantly reducing the proportion of beneficial bacteria, such as *Anaerobiospirillum*, *Butyricicoccus*, *Brevinema*, *Faecalibacterium*, *Faecalicoccus*, *Gplla*, *Lachnospira* and *Paraprevotella* (Additional file 4: Fig. S4).

### Interconnections between major flora

**Fig. 6:**
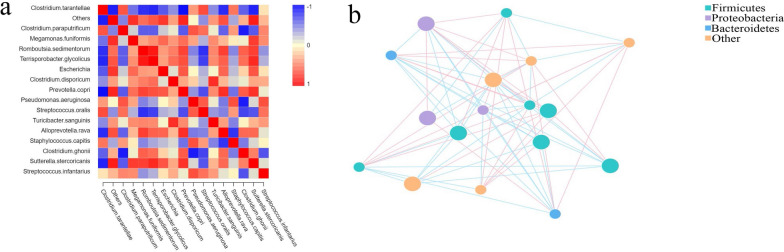
Correlation networks of the microbiome.** a** Heatmap of correlation coefficients in the CI, II and Tc groups. **b** Network map between species. Each node in the graph represents a species, with the color of the dot representing the highest relative abundance observed within these subgroups. The size of the dot corresponds to the average relative abundance of the species, with larger dots indicating higher abundance. The species are connected to each other through straight lines, with the color pink indicating a positive correlation and blue indicating a negative correlation. The thickness of the lines reflects the magnitude of the correlation, with only correlation coefficients > 0.2 between species being displayed

Construction networks revealed that the phylum Firmicutes showed relatively more numerous points and points with a larger area, meaning that the average relative abundance of the species was higher. At the same time, there was an interaction relationship between Firmicutes, Proteobacteria and Bacteroidetes (Fig. 6b). In addition, we constructed a heatmap of the correlation analysis to illustrate the correlation between species that differed at all taxonomic levels (Fig. 6a).

## Discussion

The intestinal microbiota is a complex ecosystem of trillions of microorganisms which play a crucial role in maintaining a balanced environment also regulate various physiological aspects such as metabolism, immune response, and energy regulation [15, 16]. However, infections by viruses, bacteria and parasites will disrupt this balance and have adverse effects on the host’s health. When parasites invade the intestines, they interact with the existing microbiota, which can influence their survival, reproduction and virulence [17]. *Toxocara canis*, a parasite found in dogs' gastrointestinal tracts, damages the intestinal epithelial barrier and disrupts homeostasis. As a result, researchers are increasingly interested in understanding how *T. canis* infection affects the composition of the intestinal microbiota. However, only limited research on the role of parasite-associated microbiota during infection has been performed.

In the present study, we employed sequencing of the 16S rRNA gene amplicon to examine alterations in species composition and relative abundance of the microbiota. Analysis of the data revealed several key findings. The Venn diagram, rank-abundance curve and alpha diversity analysis provided insights into microbiota diversity, richness and evenness. Notably, the Tc group exhibited the lowest levels of microbiota diversity, richness and evenness, while the II group had significantly lower levels of microbiota diversity, richness and evenness compared to the CI group. The beta diversity analysis demonstrated that microbiota species composition in the II and Tc groups were similar. Together, these results suggested that the intestinal flora abundance in dogs with *T. canis* infection was decreased, and that the abundance and diversity of flora community structures of *T. canis* were significantly lower than those of the intestinal flora of its host. More importantly, the composition of the intestinal flora of dogs infected with *T. canis* was similar to the flora carried by *T. canis*.

Characterizations at the phylum and genus levels were more complex and significantly varied between groups. At the phylum level, Firmicutes is the dominant flora in all three groups; at the same time, Firmicutes bacteria were more closely associated with other flora in the correlation network diagram. According to Rosa et al. [18], there is a positive correlation between the presence of *Ascaris* and *Necator* and the abundance of Firmicutes bacteria. Cooper et al. [19] reported that children co-infected with *T. trichiura* and *Ascaris lumbricoides* also displayed a higher proportion of Firmicutes. Previous research has shown that Firmicutes bacteria have the ability to metabolize free fatty acids and cholic acid, resulting in the production of butyrate. Butyrate is considered to be a crucial metabolite for maintaining colon health [20]. Additionally, butyrate acts as the primary energy source for colonic epithelial cells, regulates gene expression, differentiation and apoptosis of host cells and possesses anti-inflammatory properties. In our study, we found that the ratio of Firmicutes in the intestinal flora increased after being infected with *T. canis*, and this ratio was highest in the flora of *T. canis*. We speculated that the increased Firmicutes presence in the intestine is associated with *T. canis* infection and *T. canis* might play an anti-inflammatory role by regulating the proliferation, differentiation and metabolism of host intestinal cells; thus, it is conducive to the survival and invasion of *T. canis*. The authors of a recent study found that the transition from Bacteroidetes to Firmicutes is closely associated with helminth infections; this transition is believed to be linked to the establishment of an anti-inflammatory environment [13, 21]. Bacteroidetes are among the most abundant microorganisms in the intestine and play a key role in various physiological processes. These bacteria are involved in polysaccharide metabolism, contribute to the development of the immune system by promoting the growth of T cells and influence the expression of cytokines. Depletion of Bacteroidetes has been shown to have negative effects on the host. This study also demonstrated that following *T. canis* infection, Bacteroidetes levels decreased while the Firmicutes to Bacteroidetes (F/B) ratio increased. These changes might contribute to the suppression of the host's immune system development, ultimately establishing an anti-inflammatory environment in the intestine. In our results, the phylum Planctomycetes only appeared in the II and Tc groups. Previous studies have proved that Planctomycetes bacteria are able to produce antibiotics and antifungal molecules [22, 23]. Therefore, we assumed that during the course of *T. canis* infection, the high level of Planctomycetes in the host might play a role in killing *T. canis*. However, the specific function of Planctomycetes in *T. canis* remains to be further studied. Moreover, the proportion of Spirochaetes bacteria, which are involved in lipid metabolism in the intestine, was significantly reduced after *T. canis* infection.

At the genus level, our results showed that *Clostruidium* s.s. was the dominant flora in all three groups, further increasing after *T. canis* infection and that it had the highest abundance in the Tc group. Previous studies have confirmed that *Clostruidium* s.s., which is involved in amino acid metabolism, had adverse effects on the intestinal tract [24]. The relative abundance of the non-dominant *Staphylococcus* was higher in the II and the Tc groups; in comparison, this genus was almost undetectable in the CI group. *Staphylococcus* is a major opportunistic pathogen that is able to cause a wide variety of diseases in humans and animals, and it can decompose glucose, maltose, sucrose and mannitol [25]. Cooper et al. [19] detected an increased abundance of *Clostruidium* s.s. and *Streptococcus* genera in children co-infected with *T. trichiura* and *A. lumbricoides*. This is consistent with our experimental results. During *T. canis* infection, the increased levels of *Clostruidium* s.s. and *Streptococcus* allowed *T. canis* to maintain a favorable metabolic environment in the intestine. Also, *Romboutsia*, *Terrisporobacter,* and *Prevotella*, which have the function of producing short-chain fatty acids, were significantly reduced after *T. canis* infection, thus increasing the pH in the intestine, which in turn increased the abundance of harmful bacteria, such as *Streptococcus*. In addition, the proportion of harmful bacteria, such as *Akkermansia*, *Anaerococcus*, *Corynebacterium*, *Craurococcus*, *Frederiksenia* and *Haemophilus*, increased significantly, while the proportion of beneficial bacteria, such as *Anaerobiospirillum*, *Butyricicoccus*, *Brevinema*, *Faecalibacterium*, *Faecalicoccus*, *Gplla*, *Lachnospira* and *Paraprevotella* decreased significantly. More importantly, *Corynebacterium* and *Akkermansia* are highly expressed in *T. canis*. We speculated that *Corynebacterium* could carry out anaerobic metabolism to provide energy for *T. canis* growth and development, and that *Akkermansia* could use intestinal mucin as a carbon and nitrogen source for metabolism; thus, the increased abundance of these two genera could not only provide energy for *T. canis*, but also cause damage to the intestinal mucosa. While our current experimental results indicate a similarity in the microflora composition between the II and Tc groups, they do not directly prove that the changes in the host's intestinal microflora composition after infection were caused solely by the microflora carried by *T. canis*. To address this, we plan to detect the flora components of *T. canis* eggs and internal organization of *T. canis* adults in future studies, with the aim to confirm whether the changes in the host's intestinal flora are directly caused by *T. canis*.

## Conclusions

Based on an analysis of the structure and composition of the intestinal microbial community, we found that Firmicutes and Planctomycetes bacteria might cause an imbalance in the host’s intestinal homeostasis. Further analysis of the differentially expressed bacteria at the genus level revealed that some of the pathogenic bacteria, such as *Clostruidium* s.s. and *Staphylococcus* in the host’s intestine were increased. In conclusion, our finding demonstrate that *T. canis* infection could affect the composition and diversity of the host intestinal flora.

### Supplementary Information

**Additional file 1: Figure S1.** Histogram depicting the distribution of effect size for LDA.

**Additional file 2: Figure S2.** A clade map, where the center of the circle represents different taxonomic levels, ranging from phylum to genus. Each small circle within the clade map represents a classification within a specific level, and the diameter of each circle is proportional to its relative abundance.

**Additional file 3: Figure S3.** Differences in the results of the Wilcox test for harmful bacteria at the genus level. Left panel, histogram that shows the relative abundance of each group; center panel, log2 value of the average relative abundance ratio for the same taxon between two groups; right panel, display of the *P*-value and FDR adjusted significance level obtained from a Wilcoxon test. If the *P*-value and FDR adjusted significance level are < 0.05, the microbe is considered to be significantly different between the two groups.

**Additional file 4: Figure S4.** Differences in the results of the Wilcox test for beneficial bacteria at the genus level. Left panel, histogram that shows the relative abundance of each group; center panel, log2 value of the average relative abundance ratio for the same taxon between two groups; right panel, display of the *P*-value and FDR adjusted significance level obtained from a Wilcoxon test. If the *P*-value and FDR adjusted significance level are < 0.05, the microbe is considered to be significantly different between the two groups.

## Data Availability

The datasets supporting the findings of this article are included within the paper and its supplementary materials.

## Abbreviations

*T.canis*: *Toxocara canis*
*H. Taichui*: *Haplorchis Taichui*
BMI: Body Mass Index
rRNA: Ribosomal RNA
gDNA: Genomic DNA
OTU: Operational taxonomic units
LEfSe: Linear discriminant analysis effect size
LDA: Linear discriminant analysis
PAM: Partitioning Around Medoids
ANOVA: Analysis of variance
PLS-DA: Partial Least Squares Discriminant Analysis
FDR: Fssalse discovery rate
