# Supplementary material for: Toxocara canis-induced changes in host intestinal microbial communities
Source: Parasit Vectors. 2023 Dec 19;16:462. doi: 10.1186/s13071-023-06072-w (PMC10729416; doi:10.1186/s13071-023-06072-w)
Supplement: Supplementary file 1 — Additional file 1: Figure S1. Histogram depicting the distribution of effect size for LDA. [file 13071_2023_6072_MOESM1_ESM.pdf]

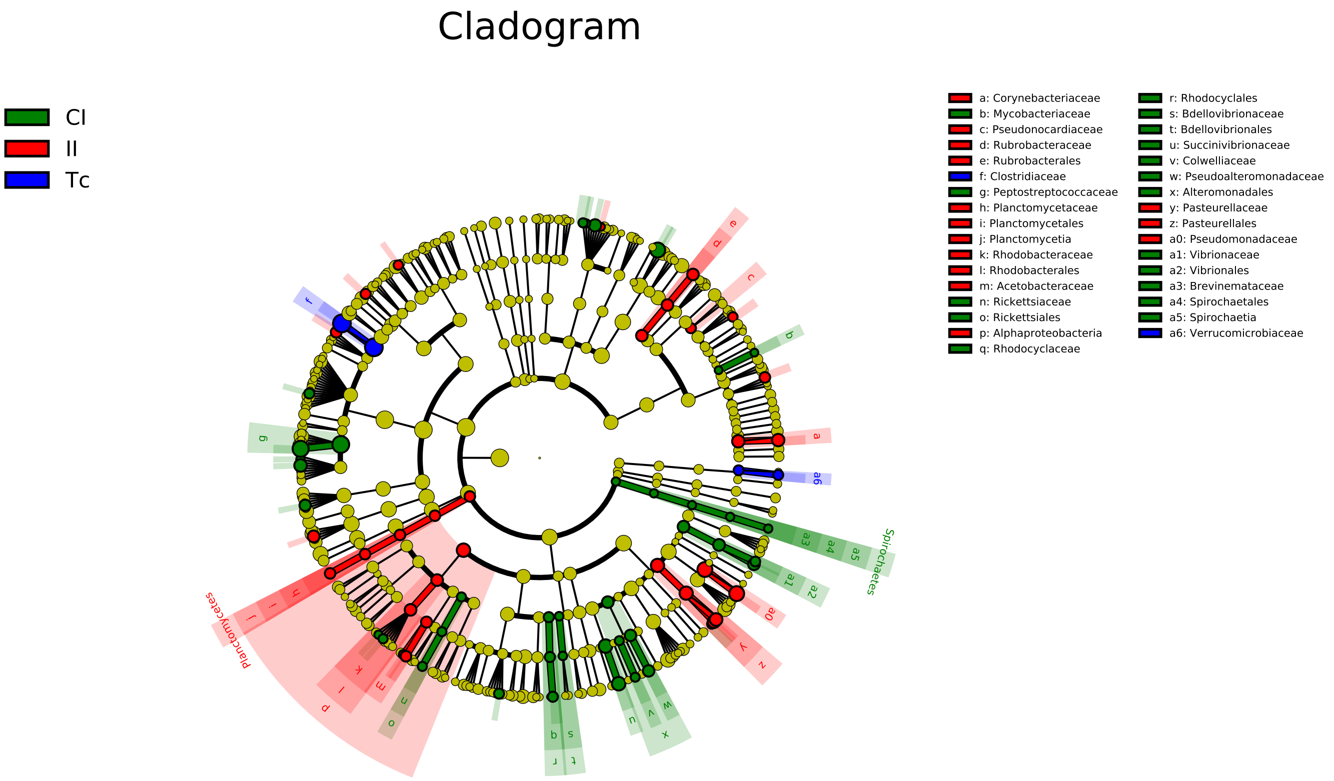

Cladogram
CI
II
Tc
a: Corynebacteriaceae
b: Mycobacteriaceae
c: Pseudonocardiaceae
d: Rubrobacteraceae
e: Rubrobacterales
f: Clostridiaceae
g: Peptostreptococcaceae
h: Planctomycetaceae
i: Planctomycetales
j: Planctomycetia
k: Rhodobacteraceae
l: Rhodobacterales
m: Acetobacteraceae
n: Rickettsiaceae
o: Rickettsiales
p: Alphaproteobacteria
q: Rhodocyclaceae
r: Rhodocyclales
s: Bdellovibrionaceae
t: Bdellovibrionales
u: Succinivibrionaceae
v: Colwelliaceae
w: Pseudoalteromonadaceae
x: Alteromonadales
y: Pasteurellaceae
z: Pasteurellales
a0: Pseudomonadaceae
a1: Vibrionaceae
a2: Vibrionales
a3: Brevinemataceae
a4: Spirochaetales
a5: Spirochaetia
a6: Verrucomicrobiaceae
Spirochaetes
Planctomycetes
