# Supplementary figures and images for: Toxocara canis-induced changes in host intestinal microbial communities
Source: Parasit Vectors. 2023 Dec 19;16:462. doi: 10.1186/s13071-023-06072-w (PMC10729416; doi:10.1186/s13071-023-06072-w)

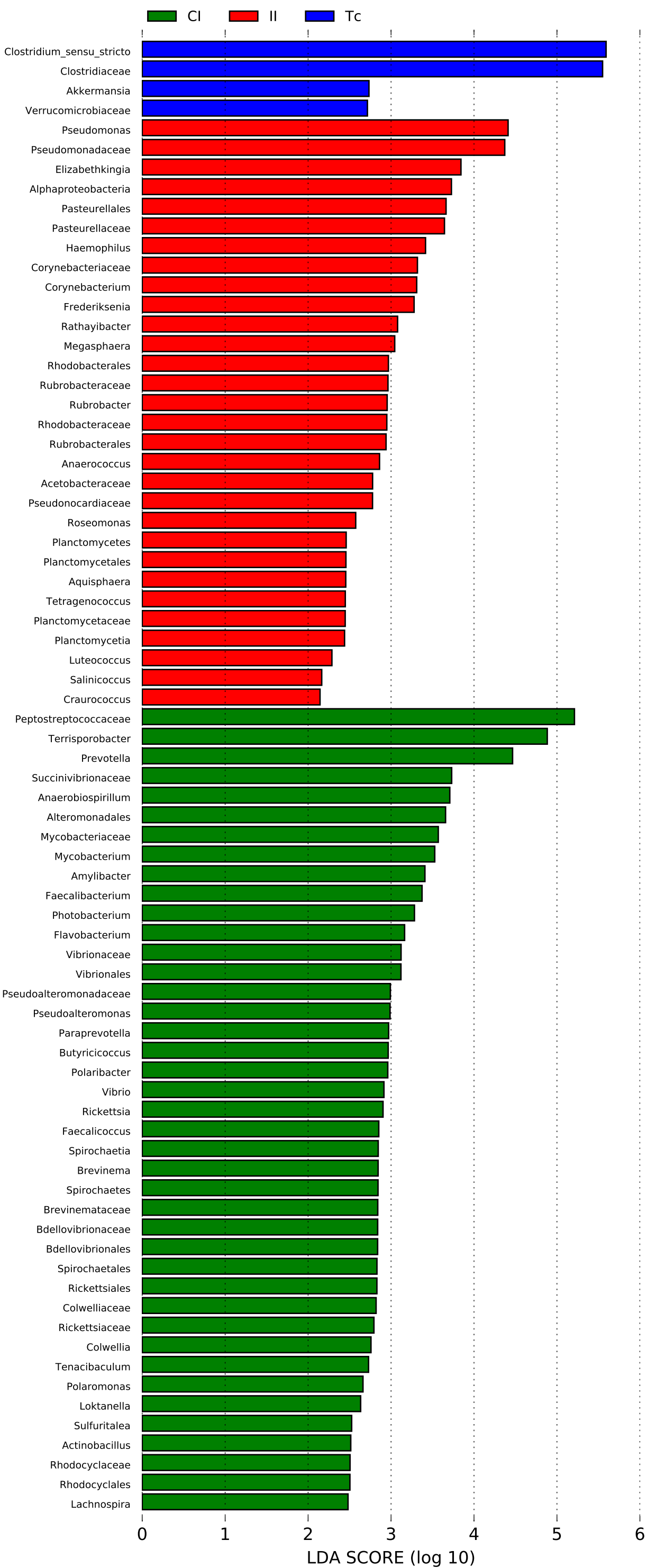

Supplement: Supplementary file 2 — Additional file 2: Figure S2. A clade map, where the center of the circle represents different taxonomic levels, ranging from phylum to genus. Each small circle within the clade map represents a classification within a specific level, and the diameter of each circle is proportional to its relative abundance. [file 13071_2023_6072_MOESM2_ESM.pdf]

**a**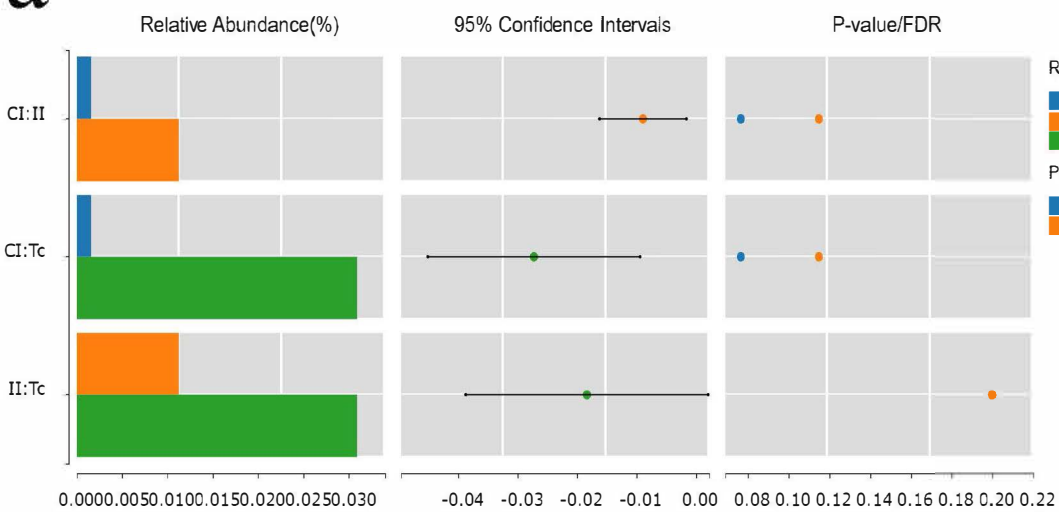**b**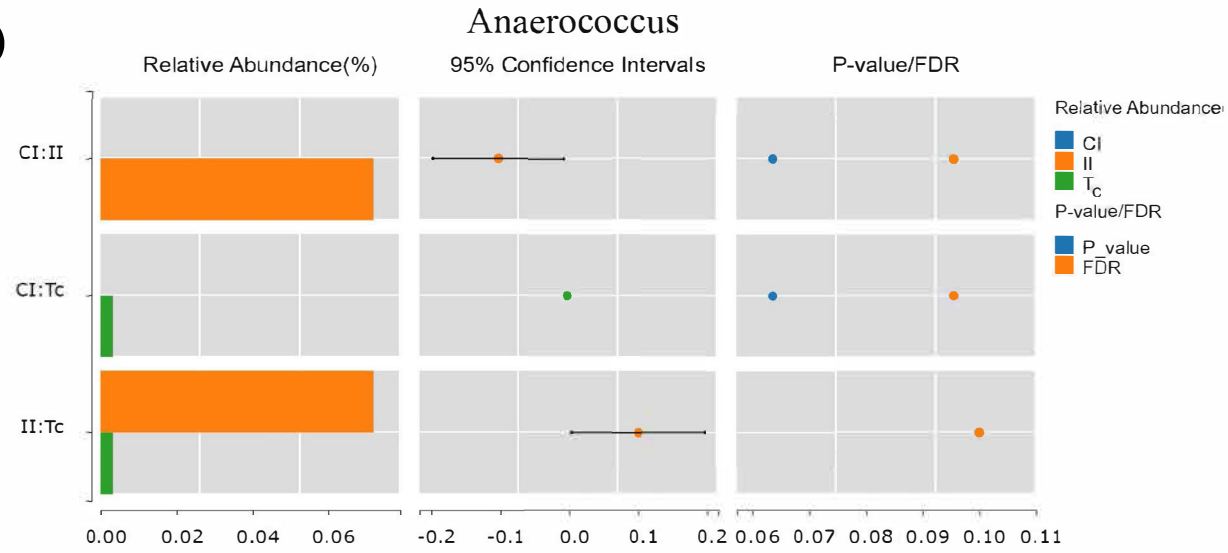**c**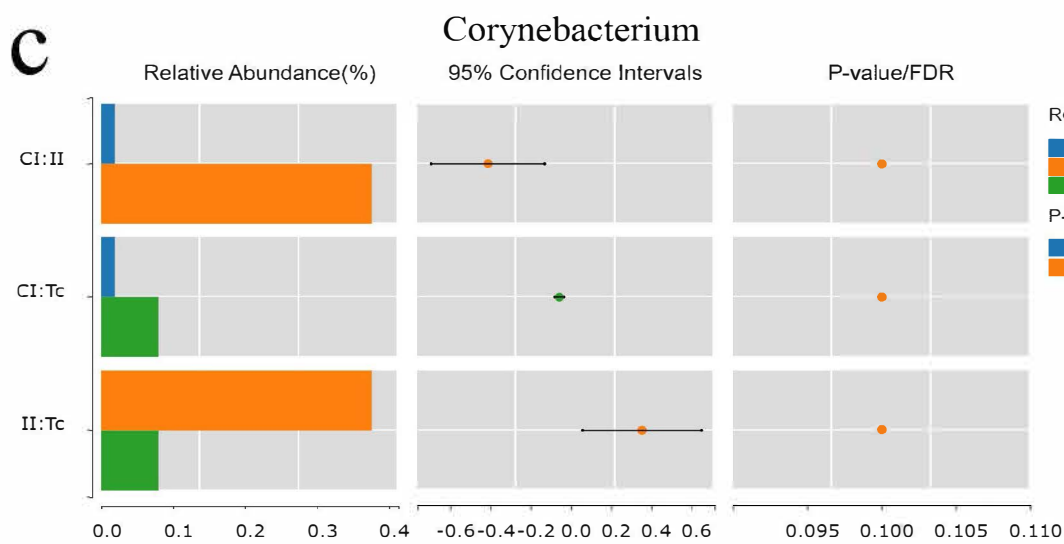**d**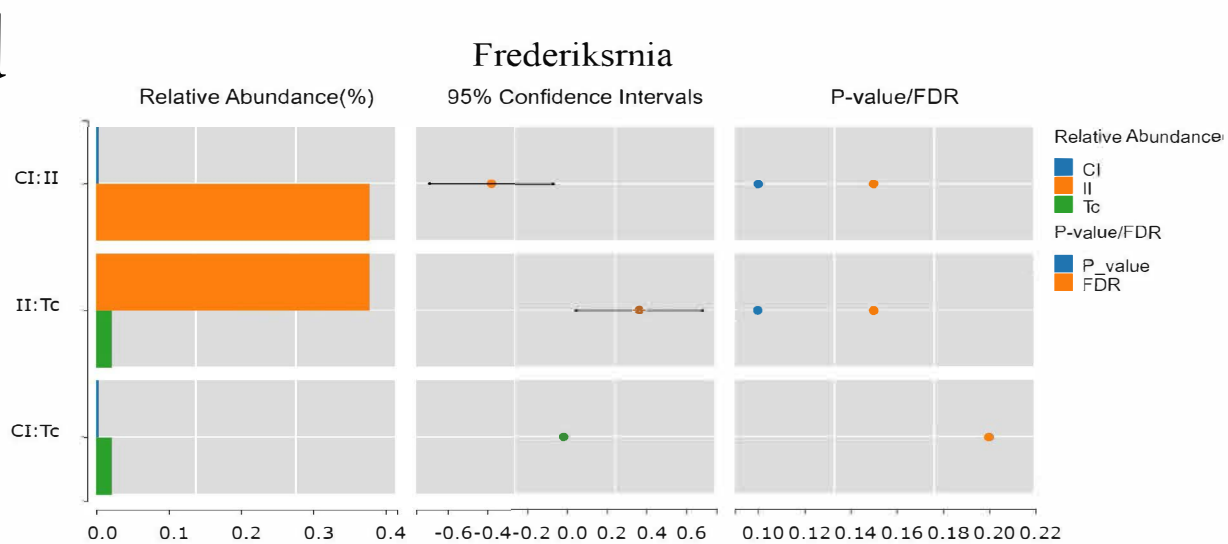**e**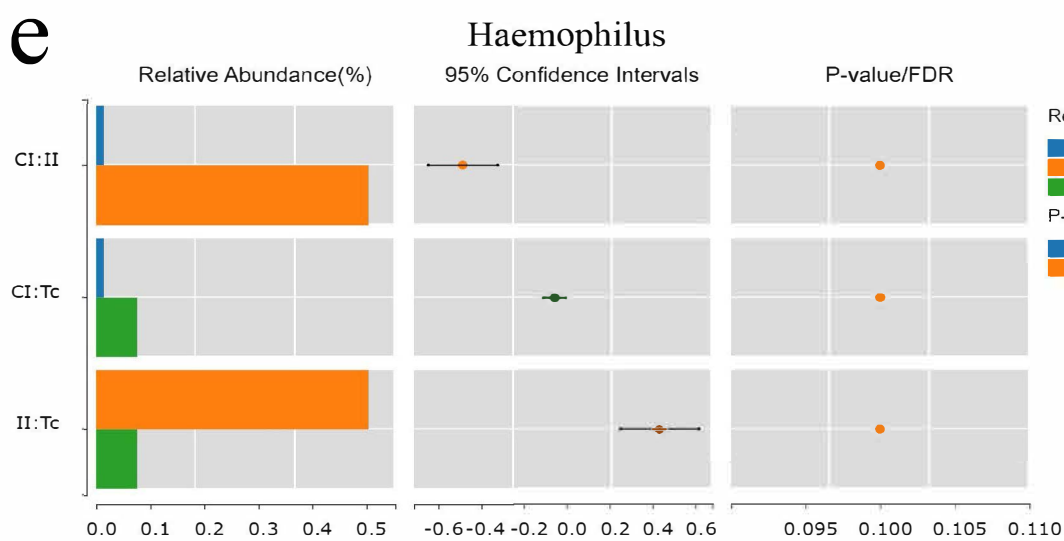**f**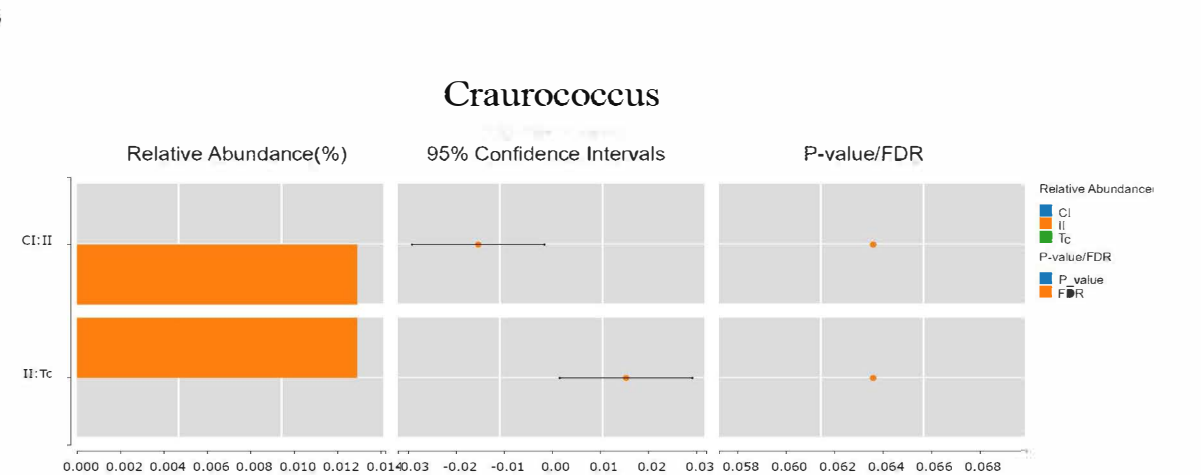

Supplement: Supplementary file 3 — Additional file 3: Figure S3. Differences in the results of the Wilcox test for harmful bacteria at the genus level. Left panel, histogram that shows the relative abundance of each group; center panel, log2 value of the average relative abundance ratio for the same taxon between two groups; right panel, display of the P-value and FDR adjusted significance level obtained from a Wilcoxon test. If the P-value and FDR adjusted significance level are < 0.05, the microbe is considered to be significantly different between the two groups. [file 13071_2023_6072_MOESM3_ESM.pdf]

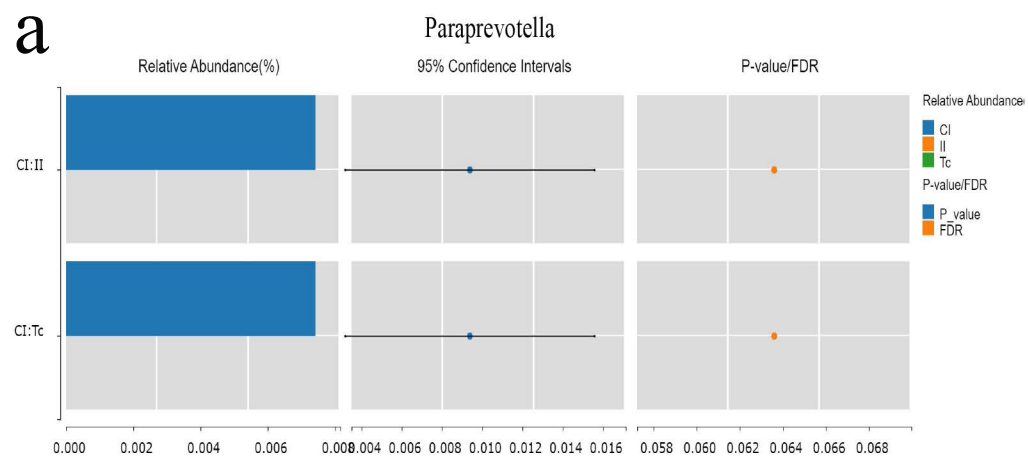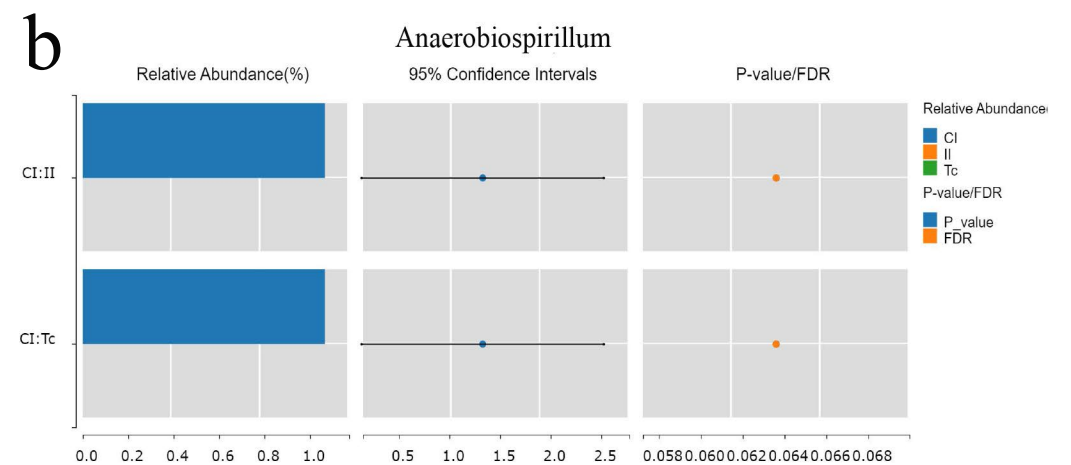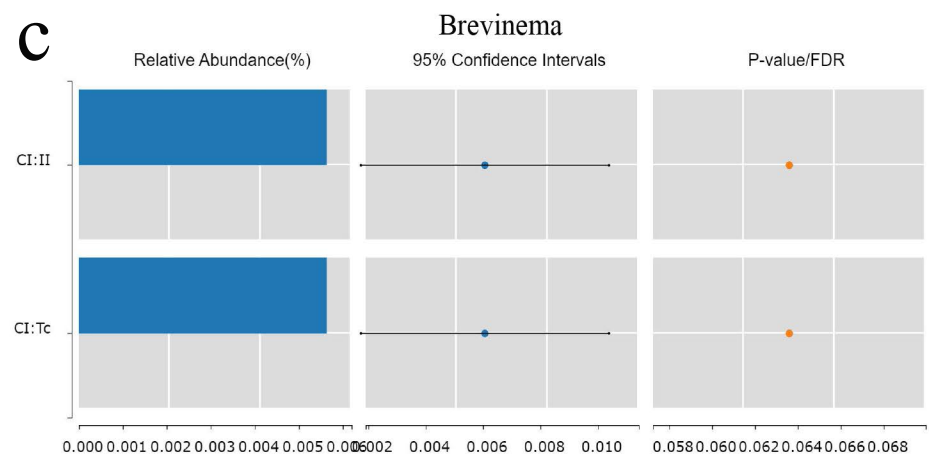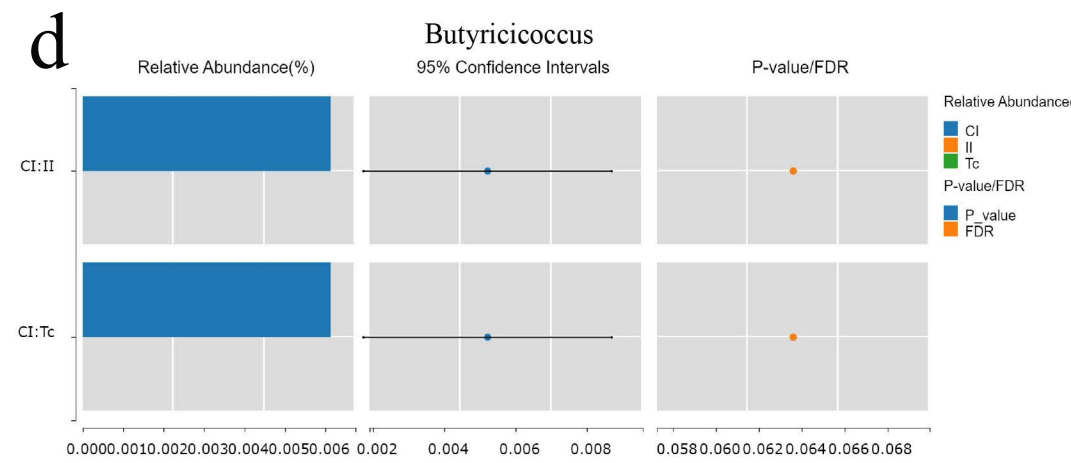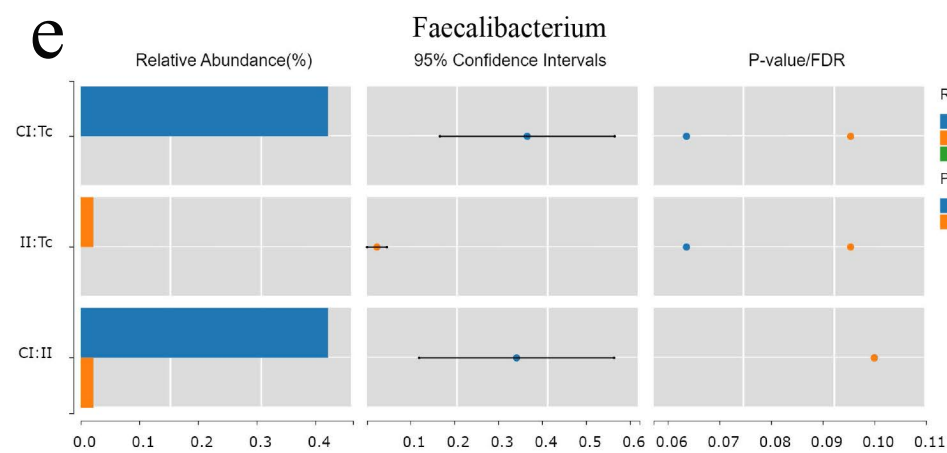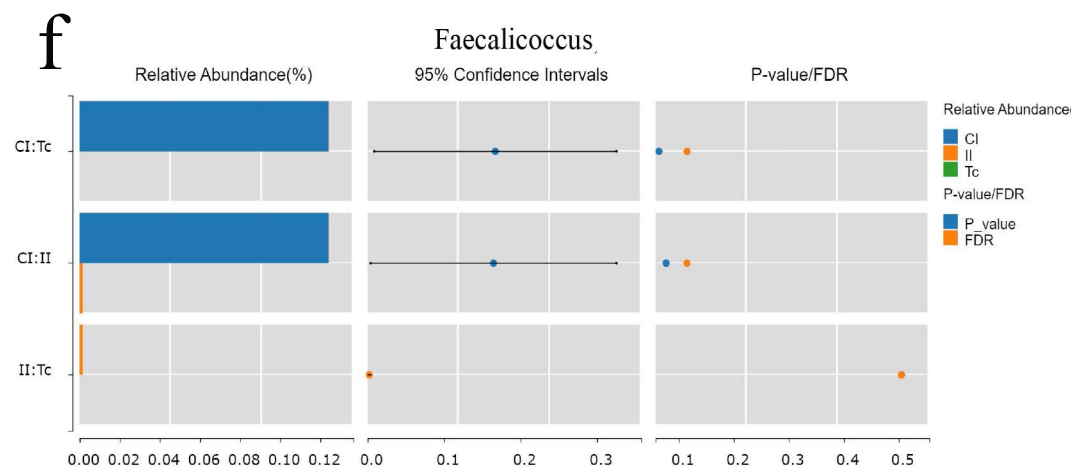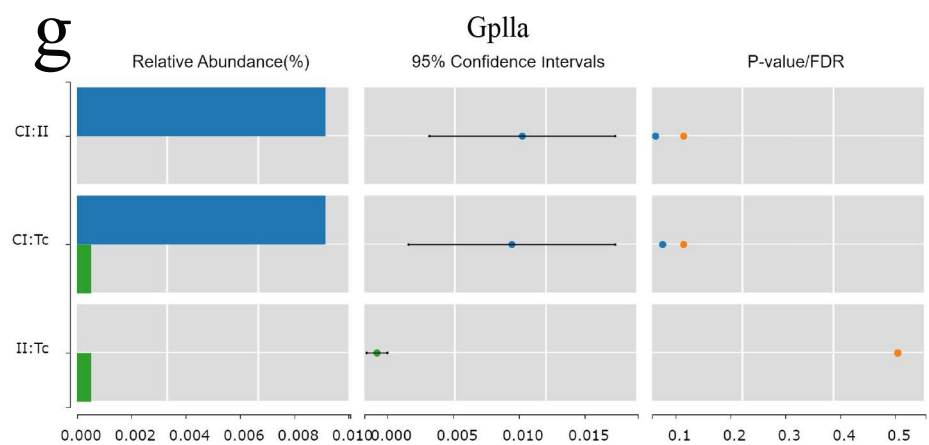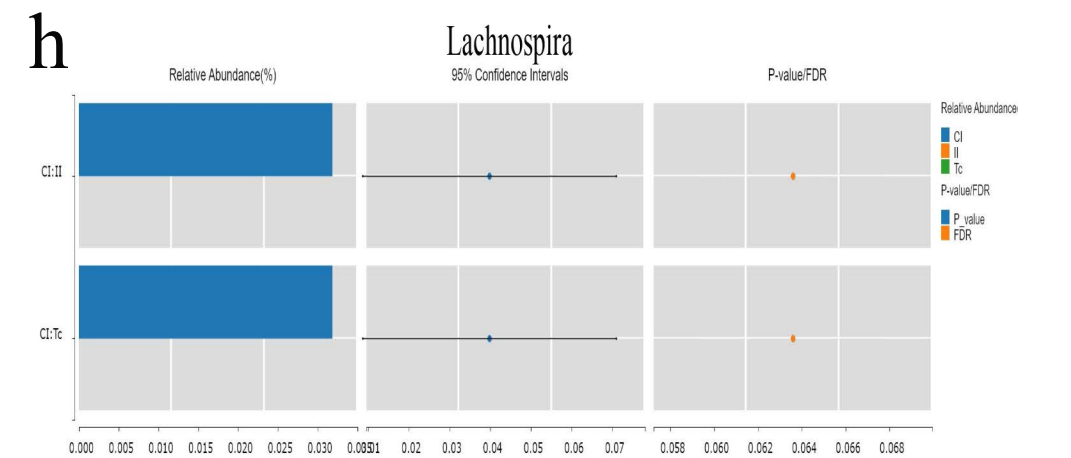

Supplement: Supplementary file 4 — Additional file 4: Figure S4. Differences in the results of the Wilcox test for beneficial bacteria at the genus level. Left panel, histogram that shows the relative abundance of each group; center panel, log2 value of the average relative abundance ratio for the same taxon between two groups; right panel, display of the P-value and FDR adjusted significance level obtained from a Wilcoxon test. If the P-value and FDR adjusted significance level are < 0.05, the microbe is considered to be significantly different between the two groups. [file 13071_2023_6072_MOESM4_ESM.pdf]
